# Guideline evaluation and implementation mechanisms in school health services (GuideMe): protocol for a hybrid randomized factorial trial

**DOI:** 10.1186/s12913-023-10179-2

**Published:** 2023-11-15

**Authors:** Åse Sagatun, Thomas Engell, Malene Brekke, Hege Sjølie, Stine M. Ekornes, Kristin Sofie Waldum-Grevboe, Kristine Pape, Kirsti Kvaløy, Annette Jeneson, Anna Stigum Trøan, Anne Liv Askeland, Line Stien, Solveig Holen

**Affiliations:** 1 Regional Centre for Child and Adolescent Mental Health, Eastern and Southern Norway (RBUP/PILAR), Oslo, Norway; 2 VID Specialized University (VID), Oslo, Norway; 3 Regional Centre for Child and Youth Mental Health and Child Welfare (RKBU Central Norway), Norwegian University of Science and Technology (NTNU), Trondheim, Norway; 4 Department of Public Health and Nursing, Faculty of Medicine and Health Sciences, Norwegian University of Science and Technology (NTNU), Trondheim, Norway; 5 HUNT Research Centre, Department of Public Health and Nursing, Faculty of Medicine and Health Sciences, Norwegian University of Science and Technology (NTNU), Trondheim, Norway

**Keywords:** Implementation strategies, Implementation mechanisms, Guideline, School health services, EPIS, Multifactorial design, Hybrid study

## Abstract

**Background:**

Norwegian school health services received a national best-practice guideline in 2017. To promote healthy life skills and identify adolescents needing support, the guideline includes strong recommendations for individual consultations with all 8th graders and increased collaboration with schools. To help implement the recommendations, a blended implementation strategy (SchoolHealth) was co-created with school nurses, students, and stakeholders. SchoolHealth consists of three implementation elements: Digital dialog and administration tool (audit and feedback +), Dialog support (external consultation), and Collaboration materials (targeted dissemination). This hybrid study will test the main and combined effects of the elements on guideline fidelity and effectiveness.

**Methods:**

The GuideMe study is a factorial cluster randomized controlled trial examining SchoolHealth's effectiveness on guideline fidelity and guideline effectiveness goals. Forty Norwegian secondary schools will be randomized to eight different combinations of the elements in SchoolHealth. Participants will include school nurses and school personnel from these schools, and 8^th^ grade students (*n* = *1200).* Primary outcomes are school nurses' fidelity to the guidelines and student's ability to cope with their life (i.e., health literacy, positive health behaviors and self-efficacy). Quantitative methods will be used to test effects and mechanisms, while mixed- and qualitative methods will be used to explore mechanisms, experiences, and other phenomena in depth. Participants will complete digital questionnaires at the start and end of the schoolyear, and after the consultation during the schoolyear. The study will run in two waves, each lasting for one school year. The multifactorial design allows testing of interactions and main effects due to equal distribution of all factors within each main effect. Sustainment and scale-up of optimized SchoolHealth elements using national infrastructure are simultaneously prepared.

**Discussion:**

The study will investigate possible effects of the implementation elements in isolation and in combination, and hypothesized implementation mechanisms. In-depth study of user experiences will inform improvements to elements in SchoolHealth. The results will yield causal knowledge about implementation strategies and the mechanisms through which they assert effects. Mixed-methods will provide insights into how and when the elements work. Optimizing guideline implementation elements can support adolescents in a crucial life phase.

**Trail registration:**

ISRCTN24173836. Registration date 8 August 2022.

**Supplementary Information:**

The online version contains supplementary material available at 10.1186/s12913-023-10179-2.

## Background

The Norwegian Directorate of Health launched a new national guideline for school health services in 2017. The guideline's aim is to promote service quality and sustainability, with less unwanted variation in practices and more coherent service pathways for students. The guideline strongly recommends individual health-promoting consultations with all 8^th^ graders, aiming to improve the students' ability to cope with life and to thrive by increasing health literacy, promoting positive health behaviors and self-efficacy, and identifying students needing follow-up. The recommendations emphasize empowering students in consultations and focusing on their needs. The guideline also strongly recommends interprofessional collaboration with schools to promote quantity and quality of care, and increase student attendance [1]. Although the guidelines are based on evidence and professional consensus [2], the effects of adhering to the guidelines have not been evaluated.

Adolescence is a crucial phase in which future life opportunities and patterns of adult health develop [3]. Therefore, adolescence is important for concurrent and prospective well-being and the economic development of nations [4]. Attending secondary school is free and obligatory in Nordic countries. Thus, schools provide opportunities to promote positive relationships, healthy behaviors, and resilience to cope with stressful events regardless of social background. The school health service is a mandatory part of the municipal health services in Norway. They are located at schools, free of cost for all students, and have health promotion and prevention as core aims [5].

The guideline recommendations are professionally normative. Any service choosing to deviate must document and justify their choice. However, the guideline is not explicit regarding *how* the school health services should implement the recommendations and reach their intended goals.

Implementation of national guidelines is a struggle across public service sectors [6]. Successful implementation and sustainment rely upon effective strategies appropriately addressing key implementation determinants and mechanisms across service levels [7, 8]. These mechanisms may be caused by dynamic connections between different elements of implementation (e.g., discrete implementation strategies, processes, and contextual circumstances; [9]), the guideline being implemented (e.g., their compatibility and relevance for practice), and the people doing and receiving implementation (e.g., the self-efficacy and capacities of practitioners). Empirical evidence about the most effective and efficient implementation strategies is scarce [7]. Also, implementation strategies are typically evaluated in packages of several discrete strategies, such as multi-element and blended strategies [9, 10]. Thus, it remains uncertain what different discrete strategies and elements contribute to effectiveness, how they contribute, and which are likely superfluous [9].

Through a human-centered co-creation approach, we developed a guideline implementation tool called SchoolHealth. The first version, inspired by a Danish equivalent named BørnUngeLiv.dk, has been found feasible and user-friendly in pilot testing [11]. Subsequently, SchoolHealth has been improved based on pilot results and re-designed into three elements representing discrete implementation strategies: (1) Digital dialog and administration tool (audit and feedback +), (2) Dialog support (external consultation), and (3) Collaboration materials (targeted dissemination). The elements represent complementary implementation strategies tailored to facilitate the implementation of the guideline with fidelity and help services reach the guidelines' intended goals. An important aspect of achieving the guideline goals is ensuring appropriate user pathways for adolescents in health services. However, how adolescents with health vulnerabilities are handled in the healthcare system is largely unknown [12], including the role of school health services in identifying follow-up needs.

### The current study

The overall objectives of the GuideMe study are to help the school health services implement the guideline recommendations and reach their goals, and simultaneously increase scientific knowledge about effective implementation strategies and health service use among students.

We will conduct a hybrid cluster randomized factorial experiment to evaluate and optimize the effectiveness of SchoolHealth. Quantitative-, qualitative-, and mixed- methods will be used to evaluate the main and combined effects of the three implementation elements on fidelity to the guideline, school and student outcomes (guideline goals), and investigate mechanisms of change and user experiences. Baseline data will be complemented with epidemiological studies and registry data to study students’ health service use in Norway. Additionally, we will prepare for system-wide scale-up of the optimized version of SchoolHealth by developing solution designs for national infrastructure.

## Methods

### Research questions

The study will investigate the following research questions:

What are the main and combined effects of the implementation elements in SchoolHealth on fidelity to the guideline recommendations for:
  - The individual 8^th^-grade consultations with students.
  - School health services collaboration with schools.
i) What are the main and combined effects of the implementation elements in SchoolHealth on:
  - Identification of vulnerable students in need of follow-up.
  - Students' health literacy, health behaviors, self-efficacy, quality of life, school environment, and attendance?
  - Students' involvement in the 8^th^-grade consultations?

- ii) How are effects associated with school nurses' fidelity to recommendations?

3. i) What are the main and combined effects of the implementation elements in SchoolHealth on:
  - Interprofessional collaboration.
  - School nurses' work-related self-efficacy and relation with students?

- ii) How are effects associated with school nurses' fidelity to recommendations?

4. Through what mechanisms do implementation elements assert their influence on implementation outcomes, and how?
  - How do individual and contextual implementation determinants influence fidelity and effects?
5. What are the participants' experiences with SchoolHealth?
  - School nurses' experiences with the elements in SchoolHealth, 8th-grade consultations, and collaboration with schools.
  - Teachers' experiences with interprofessional collaboration.
  - Students' experiences with the 8th-grade consultation and perspectives on health literacy and quality of life.
  - Experiences in Norway compared to the Danish equivalent.
6. What are the associations between self-reported health status in adolescence and user pathways in health- and welfare services﻿?

### Study setting

The study setting is Norwegian lower-secondary schools and school health services. The Norwegian school system is mainly public. The first ten years are compulsory, and all who have completed compulsory schooling are granted the right to three to four years of free upper-secondary education.

The school health service in Norway is part of the primary municipal health care services. The service aims to promote good health and prevent disease. They work on individual, group, and universal levels. The resources and structures of the services, as well as what they offer, vary substantially between municipalities [13, 14].

### Participants

**Fig. 1:**
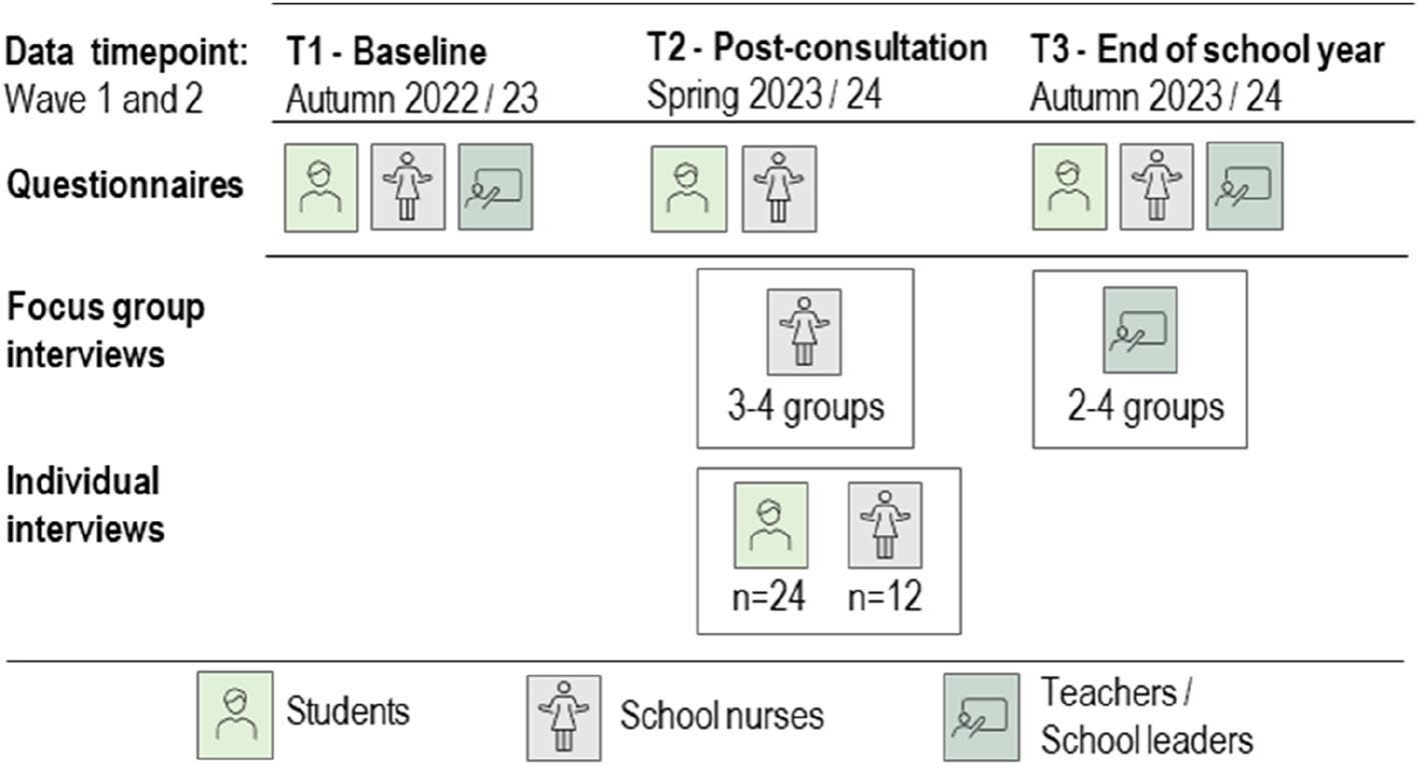
Participants and data-collection period

This multicenter study will collect data from 8^th^-grade students, school nurses, their leaders, and school personnel from several municipalities in southeast and central Norway, representing both rural and urban areas. Participating schools choose which 8^th^-grade classes (1–3 classes) to include. All students in the participating classes will then be invited. The data-collection period will last for two school years (2022/23 and 2023/24, see Fig. 1).

### Intervention: evidence-based guideline

#### 8th grade consultations

The recommendations for individual consultations with 8^th^ graders are built on legislation, evidence, and professional consensus [2]. The consultation should be health-promoting based on the students' needs, include weighing and height measuring, and address topics related to health behaviors (sleep, diet, physical activity), physical and mental health, social relationships, family, sexuality, dental health, drugs, violence, abuse, and neglect. The estimated consultation timeframe is 30 min. Before the consultation, the school nurses should familiarize themselves with the students' health records and must document the consultation in this record. The school nurse is expected to conduct follow-up consultations, initiate interprofessional collaboration, or refer students to other professionals when necessary.

#### Collaboration between school health services and schools

The guideline encompasses twelve recommendations on collaboration between school health services and schools, all marked as strong recommendations or legislative requirements [1]. The recommendations include system-oriented collaboration, monitoring students' health status, contributing to health education in groups and school classes, facilitating visits to adolescent health centers, providing health information in parent meetings, and follow-up of students' school absences. The collaboration should be systematically planned and organized.

### Implementation strategies

Table 1 specifies the experimental implementation elements per recommendations for reporting implementation strategies using the Template for Intervention Description and Replication [15]. The following describes SchoolHealth and the content of each strategy and its target functions.

**Table 1:** Specification of experimental implementation elements

Implementation strategy	Actor(s)	Actions	Action targets	Dose	Timing
Digital feedback and administration tool (DFA)—Audit and feedback +	School nurses, teachers, students	School nurses /teachers administer digital health information forms to students before consultations. Individual and group-level feedback reports about students' health, life, well-being, and needs are provided to nurses' DFA platform. The feedback provides nurses with decision support for health consultations and collaboration with schools After consultation, the students report on user satisfaction and content of consultations, including involvement and relationship with the school nurse. Group feedback reports are provided in the DFA platform	- Increase nurses’ capability, opportunity, and motivation to adhere to guidelines for consultations and collaboration with schools - Increase nurses’ self-efficacy toward consultations - Increase students involvement in and satisfaction with consultations - Identify student needs in line with guidelines - Identify school collaboration needs in line with guidelines - Nudge student follow-up in line with guidelines	Health information form is administered to all students in a class for one hour In the guideline, the consultation is estimated to take 30 min. Use of DFA is ongoing	Health information forms administered once early in the school semester. Consultations with students carried out throughout the semester. DFA is ongoing and available for nurses throughout the school year
Dialog support – Ongoing consultation	Professional school health supervisors in the research team, school nurses	- e-learning module with theory and instructional videos about effective health-promoting consultations following guidelines - Hybrid format training and consultation in conducting 8^th^-grade consultations in line with guidelines - Modelled after Reflexive teams (ref)	- Increase nurses’ capability, opportunity, and motivation to adhere to guidelines for 8^th^-grade consultations - Increase nurses’ self-efficacy toward 8^th^-grade consultations - Provide a space for case reflections, problem-solving, and collegial support	3 × 180 min, 1 × 60 min, 6 e-learning videos of 7–17 min, e-learning approx. 60min reading time	Early in the semester, mid semester, late semester, and a closing reflection session after the semester
Collaboration materials – Active dissemination of materials	Research staff disseminates material. Schools and school health services are encouraged to plan and conduct collaboration meetings	- Disseminate digital educational and organizational materials that can be used by school personnel and health nurses to plan and organize collaboration - The materials are: four short videos and reflective questions targeting different topics important for interprofessional collaboration - A final meeting to exchange experiences and plan further collaboration	- Improve and structure interprofessional collaboration - Provide a space to reflect together on common goals and work - Gain better knowledge about each other’s guidelines and responsibilities - Ensure that the school and school health services know about each other’s work and collaborate on an individual and system level - Prevent silo-mentality - Plan shared work	The material encourages 5X60 minutes collaboration meetings carried out by each school and school health service. Four videos of about 15 min	Dissemination at the start of the school year, and five meetings spread out across the school year is encouraged

#### Audit and feedback + (Digital Feedback and Administration Tool [DFA])

DFA is the only element that involves the students directly. School nurses/teachers administer a digital health information form to students before their 8^th−^grade consultation. The topics in the form are based on the recommended topics in the guideline. Filling out a health form may empower students through preparing topics they can bring up in the consultation and the opportunity to reflect upon topics important to them. The school nurse will receive an *individual feedback report*, with the main aim to support school nurses in tailoring consultations to individual needs, thus increasing nurses' capability, opportunity, and motivation to use the 8^th^-grade consultation recommendations. It may also help identify students in need of additional support and follow-up. After the 8^th^-grade consultation, the students and the school nurses answer questions regarding the consultation. The students report user satisfaction, including relation with the school nurse and involvement in consultation, generating the *user satisfaction report*. The school nurse and the students answer questions about the content of the consultation (fidelity to guideline), generating the *8*^*th*^*-grade consultation report*. Both reports will be available for the school nurse and their service leaders on the DFA platform. Additionally, the DFA platform include a *school report*, an aggregated summary based on all the students' pre-consultation health information forms. Here, school health nurses and leaders can compare their schools/districts aggregated answers with that of others in the study. The school report covers topics highlighted in the guideline recommendations on which the school health service and schools should collaborate.

#### Ongoing consultation (Dialog Support)

Dialog Support aims to increase school nurses' capability, opportunity, and motivation to adhere to the guideline recommendation for 8^th^-grade consultations with fidelity and to increase their self-efficacy toward this consultation. The strategy includes an e-learning module and ongoing consultations. The e-learning has two main sections on theory and instructional videos about conducting health-promoting 8^th^-grade consultations. The first section provides health-promoting theories (salutogenesis, health literacy, empowerment, and user participation). The next section encompasses communication (starting, conducting, and ending conversations, [16]). The ongoing consultations will include training in conducting and reflections about the 8^th^-grade consultations. The consultations will be modeled after the Reflexive teams approach [17], aiming to provide a space for case reflection, problem-solving, and collegial support. They will be conducted in-person and digitally (hybrid format) and consist of four meetings.

#### Targeted dissemination (Collaboration Materials)

Collaboration Materials is the only element that involves school personnel directly. The element is labeled an active dissemination strategy because a web-based package with educational and organizational materials and resources is actively provided to teachers, head teachers, and school nurses. The main aim of the dissemination is to improve and structure interprofessional collaboration.

The element consists of four digital modules with short educational videos, reflection tasks, and a final summary module. The element will be introduced at a kick-off meeting, and participants will be encouraged to plan how to implement the modules during one school year. The project staff does not engage in this.

Each module is estimated to last approximately 60 min, and the topics are based on what is considered important to promote interprofessional collaboration and support [18]. The topics are: (1) Conditions for systematic and interprofessional collaboration at different levels of intervention (2), Overlapping topics in the National curriculum for schools and the Guideline for school health services, (3) Available resources and supports for evidence-based interventions and utilization of existing data, and (4) Interprofessional communication – identification of barriers and facilitators, (5) exchange experiences and plan further collaboration.

### Design

The study is a hybrid type 2 trial, studying both fidelity to guidelines and guideline effects using: (i) a cluster randomized factorial experiment, ii) hermeneutic phenomenological qualitative methods, and (iii) convergent and sequential mixed-methods. To study health service use in Norwegian adolescent, survey data will be linked with national registers (iv)*.*

#### The randomized factorial experiment (i)

We will employ a stratified, randomized cluster factorial design to evaluate the effects of the three implementation elements separately and in different combinations. The schools will be randomized to one of eight different experimental conditions.

The factors in Table 2 reflect the three previously described elements. The three elements are complementary, and together (yes/yes/yes) represent a blended implementation strategy that, in theory, should elicit the strongest outcomes based on an additivity or ecology principle (i.e., effects of implementation strategies are the sum of their parts or more than the sum of their parts, [9]. However, these implementation elements have rarely been evaluated independently or together. The multifactorial design allows for the testing of interactions and main effects due to the equal distribution of all factors within each main effect.

**Table 2:** Experimental conditions in the factorial experiment

Experimental condition	SchoolHealth—Implementation elements		
	1. Digital feedback and administraton tool	2. Dialog support	3. Collaboration materials
1	Yes	Yes	Yes
2	Yes	Yes	No
3	Yes	No	Yes
4	Yes	No	No
5	No	Yes	Yes
6	No	Yes	No
7	No	No	Yes
8	No	No	No

##### Statistical methods (i)

Primarily, linear mixed-effects models will be used to investigate the implementation elements' main- and interaction effects on fidelity to the guideline recommendations and guideline goals. In addition, a stepwise theory-informed strategy will be used to explore the effect of implementation determinants.

To investigate psychometric properties, exploratory and confirmatory factor analyses will be performed on instruments with sufficient respondents (primarily instruments administered to students). For all instruments, correlations between subscales will be computed using Pearson's r and other relevant statistics. Internal consistency for the scales and subscales will be investigated using Cronbach's alpha and other relevant statistics.

#### Hermeneutic phenomenological qualitative methods (ii)

We will use a hermeneutic-phenomenological qualitative approach [19] to explore the experiences of students, school nurses, and school personnel. We use hermeneutic phenomenology to explore and interpret phenomena as understood and formulated by the participants [20, 21].

Qualitative individual and focus group interviews will be used to gain in-depth knowledge of central phenomena and user experiences [21, 22]. Qualitative analyses of meaning content will be carried out by informed models for qualitative analyses as described by e.g., Kvale and Brinkmann [21], Van Manen [20], and Braun and Clarke [23].

##### Qualitative interview

All interviews will be semi-structured. Guides for individual interviews with students and school nurses contain 4–5 predetermined themes with follow-up questions, and permit an open dialog. Students are interviewed about their experiences with the 8^th^-grade consultation, relation to the school nurse, health literacy, quality of life, and coping. School nurses' individual interviews touch upon their experiences with coping during the 8^th^-grade consultation, student relations, and how to identify and follow up students with additional needs.

The guide for focus group interviews with school nurses is made with the opportunity to add themes related to implementation after preliminary quantitative data analyses (see mixed-methods). The complete interview guide covers themes such as experiences with the 8^th^-grade consultation, collaboration with school, national guidelines, implementation of the elements, and determinants for implementation. School personnel are interviewed on three main topics regarding the nature, content, and quality of collaboration with school health services. In addition, they are asked about experiences with implementing SchoolHealth and relevant implementation determinants.

#### Mixed-methods (iii)

A mixed-methods experimental design (convergent and sequential [24]), from a pluralistic and meta-paradigmatic perspective [25], will be used to investigate experiences with the different implementation elements and the complexity of implementation mechanisms across conditions. We will corroborate quantitative and qualitative data on the value of, and experiences with, the implementation elements and the guideline recommendations. Qualitative data will provide a more in-depth understanding of findings from different viewpoints. According to Teddlie and Tashakkori [26], mixed methods research involves seeing qualitative and quantitative data as two different ends of a continuum, where one moves seamlessly across it to pursue optimal answers to the different research questions of the study. This means that the different data sources will be given different weights and priorities throughout the analytical process to best answer the research question. Thus, the sequential dimension of the design will include conducting preliminary analyses of quantitative data about implementation determinants, fidelity, and collaboration after each data collection wave to inform themes and questions for the qualitative interviews.

#### Student data and national data registers (iv)

We will compare baseline data from GuideMe on health and health service use with cross-sectional data from wave 4 of the adolescent part of the Nord-Trøndelag Health Study (Young-HUNT Study) [27], a Norwegian population-based study in Central Norway [28, 29]. In the Young-HUNT wave 4 survey there were 8066 (76% of the invited) 13–19-year-old participants. Data from GuideMe and Young-HUNT4 will also be linked with national registry data on healthcare service use, such as The Norwegian Registry for Primary Health Care (KPR) and the Norwegian Patient Registry (NPR) [30].

Health and health service use among participants in both studies will be assessed and compared. Subgroup analyses will be conducted to test whether demographics (e.g., gender, socioeconomic status, schools) affect associations between health measures (e.g., mental health, medical conditions, health behavior).

### Outcomes

**Fig. 2:**
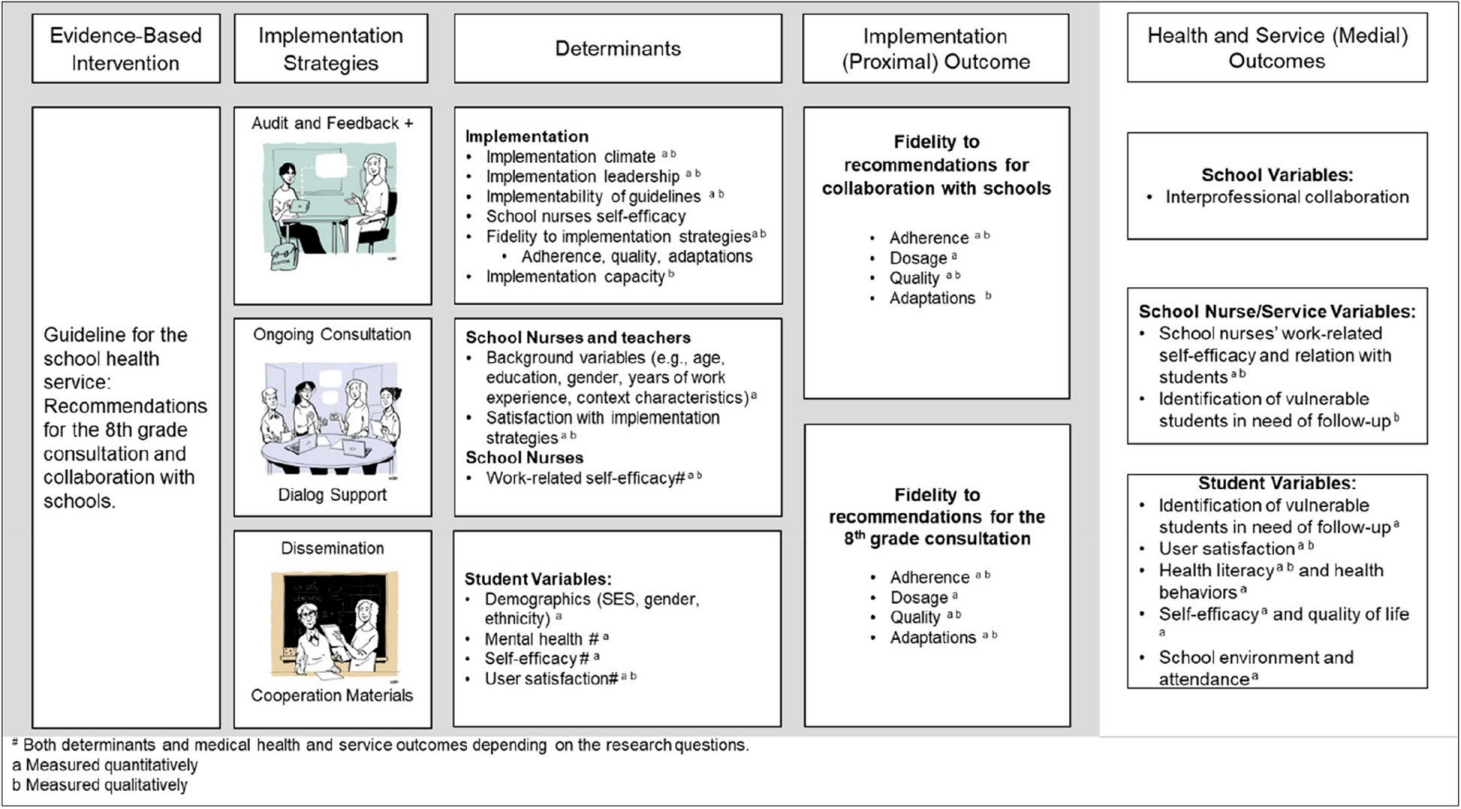
Logic model depicting a simplification of the theorized relationships between the guideline recommendations, implementation elements, determinants, and proximal and medial outcomes

The study uses the Exploration, Preparation, Implementation, and Sustainment Framework (EPIS) [31] as a theoretical framework for investigating implementation as multilevel processes influenced by innovation factors, the outer and inner implementation context, and the interplay between factors (i.e., bridging factors) across four phases of implementation [32]. Due to the highly autonomous and individual nature of school nurses' practice settings, we complement EPIS with the Capabilities, Opportunities, and Motivation model of Behavior change (COM-B) [33]) to inform explorations of how and why implementation strategies influence school nurses' fidelity to guidelines. EPIS and COM-B have informed the development of studies' theories of change and hypotheses for how the implementation strategies influence fidelity to guidelines and guideline effects (see Fig. 2 for logic model). EPIS has informed measurements of organizational and individual-level implementation determinants and focus group interviews. COM-B has informed measures of individual-level determinants of behavior change and individual interviews.

The outcomes are operationalized and described in Table 3 by measurements, data collection method, informant, and timepoint. Details about the measurement instruments, including psychometric properties and validations, are in Supplementary file 1.

**Table 3:** Outcomes and measures, measurements, method of data collection, informant, and timepoint for each measure in the factorial experiment

Outcome/measure	Measurement	Method of data collection ^a^	Informant ^b^	Timepoint		
				**T1**	**T2**	**T3**
**Implementation Outcome**						
*Fidelity to guidelines – 8*^*th*^*-grade consultation*						
Adherence to guidelines	Self-developed	Quant	N, S		x	
Dosage	Self-developed	Quant	N		x	
Quality in using guidelines	Self-developed	Quant + Qual	N, S		x	x
Adaptations to guidelines	Self-developed	Quant + Qual	N		x	x
*Fidelity to guidelines – Collaboration, schools and school health service*						
Collaboration-adherence	Self-developed	Quant	N, T	x		x
Dosage	Self-developed	Quant	N	x		x
Quality in collaboration	Self-developed	Quant	N, T	x		x
Adaptations to guidelines		Qual	N			x
**Health and Service Outcomes**						
*Identification of vulnerable students*		Qual	N			
Mental health*	SDQ	Quant	S	x		x
Student function	Self-developed	Quant	N		x	
Follow-up group	National classification		N		x	
Follow-up consultation(s)	Self-developed	Quant	N			
Somatic symptoms	CSSI-8	Quant	S	x		x
Quality of Life	KIDSCREEN-27	Quant	S	x		x
Self-efficiacy*	GSE-5	Quant	S	x	x	x
Health literacy	HLSAC	Quant + Qual	S	x	x	x
Health behaviors	Self-developed	Quant	S	x		x
School environment and attendence	Self-developed, items from similar studies	Quant	S	x		x
User satisfaction*	Partly self-developed	Quant + Qual	S		x	
Work-related self-efficiacy*	GSE-5, slightly adjusted	Quant + Qual	N	x		x
Interprofessional collaboration	Interprofessional collaboration	Quant	N, T	x		x
**Determinants**						
Implementation climate	ICS	Quant + Qual	N, L	x		x
Implementation leadership	ILS	Quant + Qual	N, L	x		x
Implementability of guidelines	FIM, AIM, IAM	Quant + Qual	N, L	x		x
Fidelity to implementation elements (adherence, adaptations, and quality)**	Self-developed	Quant + Qual	N, L, P	**	**	**
Implementation capacity		Qual	N	x		
Satisfaction with implementation elements	Self-developed	Quant + Qual	N, T, S			x
Background variables	Self-developed	Quant	N, T	x		
Demographics	Self-developed	Quant	S	x		

*Abbreviations*: *SDQ* Strengths and Difficulties Questionnaire, *CSSI-8* Children’ Somatic Symptoms Inventory, *GSE-5* General Self-Efficacy Scale, *HLSAC* Health Literacy for School-Aged Children, *ICS* The Implementation Climate Scale, *ILS* The Implementation Leadership Scale, *FIM* Feasibility of Intervention Measure, *AIM*  Acceptability of Intervention Measure, *IAM* Intervention Appropriateness Measure

^*^Both determinant and health/service outcome

^**^ Fidelity to implementation elements is monitored throughout the study

^a^Quant: Quantitative digital survey. Qual: Qualitative interviews

^b^N = School nurse, L = Leaders school health services, T = Teachers/School leaders, S = Students, P = Project staff

#### Implementation outcomes (proximal)

Fidelity to guidelines will be assessed through items encompassing the three constructs adherence to guidelines, adaptations to guidelines, and quality in using guidelines. *Adherence to guidelines* is conceptualized as adhering to specific key recommendations for how to carry out the 8^th^-grade consultation and collaboration with schools. Measurement of 8^th^-grade consultation adherence include items about whether health information was adapted to the student, and focus on habits important to promote good health. Additionally, checklist items about themes addressed and registering height and weight also index adherence. Collaboration adherence is measured with questions about how the collaboration is organized (formal and informal meetings), whether school nurses participated in any of the schools' planning hours or meetings, and topics on which the school and school nurses are supposed to collaborate and how they collaborate.

The *dosage* of 8^th^ grade consultation will be measured by the time used on the consultation, and the dosage of collaboration by the number of scheduled meetings.

*Quality in using the 8*^*th*^*-grade consultation guidelines* is indexed by post-consultation measures of school nurses’ and students’ perceptions of their alliance and achievement of the guidelines' core functions, such as empowerment, reinforcement of positive health behavior, and identification of follow-up needs. *Quality in using collaboration guidelines* is indexed by measuring perceptions of achievement of the core functions of collaboration guidelines, such as common values and understanding, role and responsibility clarification, ease of contact with each other, knowledge about each other's competence and regulations, mutual respect, and structure.

School nurses report *adaptations to consultation* through open-ended questions in the post-consultation questionnaire (T2). Adaptations will be retrospectively coded by using the Framework for Reporting Adaptations and Modifications to Evidence-based Implementation Strategies [34], labeled fidelity-consistent (positive) or fidelity-inconsistent (negative). The labeling will be based on a qualitative judgment of whether the adaptation was likely to maintain the core function of the recommendation in our theory of change (fidelity-consistent) or not [35]. The qualitative judgment will also be informed by the measures of quality. Qualitative interviews with school nurses and school personnel will explore adaptation to collaboration guidelines.

#### Health and service outcomes (medial)

The effectiveness of SchoolHealth on guideline recommendation goals will be measured through students' health and service outcomes (Table 3) [36–38] relevant to the guideline goals.

*Identification of vulnerable students* in need of follow-up will be captured qualitatively and assessed quantitatively through school nurses' evaluation of students' physical, psychological, and social functioning, registration of follow-up group, the number of follow-ups during the school year, and the student's self-reported mental health [The Strength and Difficulties Questionnaire (SDQ)] [39].

Students' health outcomes will be assessed by *somatic symptoms* (The Children’s Somatic Symptoms Inventory) [40], *quality of life* (Kidscreen-27) [41, 42], *general self-efficacy* [General Self-Efficacy scale (GSE-5)] [43, 44], and *health literacy* (Health Literacy for School-Aged Children) [45] at the start (T1) and end of the school year (T3). Health literacy and self-efficacy will also be measured post-consultation (T2), and health literacy will be explored qualitatively. Students will assess their *health behaviors* through items on behaviors of sleep, physical activity, nutrition, and screen time activities.

Students' assessments of *School environment and attendance* will be measured through a mix of self-developed questions and questions used in similar studies ([46], see Supplementary file 1 for details).

*User satisfaction* is an overall assessment of students' experiences (qualitatively) and degree of user satisfaction and empowerment in consultation (quantitatively). It includes items of involvement in consultations (like being heard and talking about what matters to them) and student-school nurse alliance, informed by both students and school nurses. The items are partly self-developed, inspired by similar scales [47, 48].

School nurses and school personnel will assess *interprofessional collaboration* between the school and school nurse [49]. School nurses will complete an assessment on their work-related self-efficacy using an adjusted version [50] of the GSE-5 [44].

#### Determinants

*Implementation determinants* will be measured to investigate their influence on fidelity to guidelines and guideline effects. These include school nurses' and leaders' assessments of implementation climate (Implementation Climate Scale) [36–38, 51], implementation leadership (Implementation leadership Scale) [52, 53], implementability of guidelines (Feasibility of Intervention Measure, Acceptability of Intervention Measure, Intervention Appropriateness Measure) [54], fidelity to implementation elements, implementation capacity (qualitative interviews), and school nurses' work-related self-efficacy [50].

*Background variables* will be collected from school nurses and teachers regarding age, gender, education, and years of work experience. Additionally, context characteristics will be assessed by school nurses. Students' assessment of *demographics* includes items on socioeconomic status, gender, and ethnicity. Other student-determinants will be assessed by mental health (SDQ) [39], self-efficacy [44, 55], and user satisfaction as described under Health and Service Outcomes.

#### Health data and linkage with national registers

Student questionnaires in GuideMe and the Young-HUNT4 Survey cover overlapping topics and identical instruments, subscales, or items. Both include for example the SDQ, items about general health and quality of life, health care use, and health behavior.

From the national registers, data on socioeconomic status, along with use of the school health services (KPR), general practitioners (KPR), physiotherapists (KPR), and specialized healthcare services (including psychiatric care) (NPR) will be linked to GuideMe data.

### Recruitment

The schools and school health services will be invited mainly through a convenience sampling approach.

#### School health services

Recruitment of school health services will be done through oral and written information and meetings with the leaders of the services. Additionally, written information will be provided to administrative leaders of the local municipalities.

#### Schools

Two different approaches will be used to invite schools: (1) After the school health services have agreed to participate or (2) simultaneously. A brief description of the study will be sent to the school leaders, with an invitation to attend an information meeting. The study will then be presented to the school principals and the school health services in each location. The interested schools will be asked to nominate a key contact person. School health services and schools agreeing to participate sign a cooperation agreement.

#### 8th grade students

Students in the participating classes and their parents will be introduced to the study via class visits by school nurses and parent meetings. The schools will provide parents with written information and a link to a digital informed consent form, including a voluntary option for providing the second parents' e-mail so that s/he can get information that the parent has consented to the student participating in the study. For students to participate in the study, at least one of the parents must complete an electronic consent form. The students will be given age-appropriate written and animated information at school. The students will consent to participate by filling in the questionnaire. A project webpage (https://guideme.rbup.no/en) is developed to enhance communication with all participants.

The recruitment of participants will be reported per the Consolidated Standards of Reporting Trials (CONSORT) guidelines for clustered randomized trials.

### Inclusion and exclusion criteria

Inclusion criteria are students who agree to participate, have informed consent from one of their parents, and are able to answer the web-based questionnaires.

The main exclusion criteria are intellectual disability or language problems, defined as not being able to complete the questionnaires. In addition, long-term school absenteeism may also be an exclusion criterion but will be considered individually. The reasons for exclusion will be documented in the CONSORT flowchart.

### Randomization in the factorial experiment (i)

The schools will be randomly assigned to test different combinations of the three implementation elements in SchoolHealth. The school randomization procedure will be carried out in R using a function specifically written for the GuideMe study. The function is developed by a statistician in collaboration with key personnel in the project and will be witnessed by an objective third party. The schools will be randomized to one of the eight experimental conditions (see Table 2).

### Power analysis and sample size in the factorial experiment (i)

An R-package called MOST developed for power analyses in factorial trials will be used (see supplementary file 2 for R-script). When conducting a factorial trial, one option for specifying effect size for power calculation is deciding the smallest effect of practical interest [56]. This can be decided using Cohen's rule of thumb [57].

We selected the following statistical attributes: α = 0.05, an effect size of d = 0.30, and statistical power of 0.80 (β = 0.20). Being a cluster trial, the design effect may affect our power calculation. Thus, an intraclass correlation coefficient (ICC) of 0.05 and an average size of clusters = 30 (SD = 15) was also accounted for [58]. The results from the power calculations indicated that 36 schools and 1080 students were needed in the study. To account for possible dropout and the need for subgroup analyses, we aim to recruit approximately 40 schools and 1200 students.

### Participants in qualitative interviews (ii and iii)

The qualitative data will be collected in both waves (Fig. 1). We will conduct individual interviews with 24 students and 12 school nurses, and focus group interviews with 12–24 school nurses, 12–24 teachers, and 6–12 school leaders. Variations in the experimental condition and geographic region will be emphasized when inviting participants to facilitate representativeness. The selection of students for qualitative interviews will be stratified [59]. When schools are selected, school nurses will provide names for students that fit pre-defined criteria regarding gender (boys/girls), quality of conversation in 8^th^-grade consultation (good/difficult), and cultural background (Norwegian/second culture).

School nurses, school personnel, and school leaders will be recruited through purposive availability sampling, emphasizing the participants` ability to elucidate a specific theme [60]. All participating nurses will be invited due to the limited number of participants and the large number of conditions. School personnel and leaders will be recruited to ensure representativeness to different experimental conditions, particularly element 3, Collaboration materials, due to their active role in this condition.

In Denmark, interviews with school nurses and teams implementing BørnUngeLiv.dk will be conducted. The main aim is to compare SchoolHealth with the Danish equivalent.

All interviews will be digitally audio-recorded and transcribed verbatim.

### Implementation of schoolhealth

#### Quality assurance/Monitoring

We will monitor implementation quality by measuring implementation fidelity to ensure validity in experimental conditions. We conceptualize implementation fidelity similarly to guideline fidelity [25]. Measures of implementation fidelity are designed to index whether implementation in each condition is conducted as planned (e.g., content, structure, dosage, materials, absentees, turnover), whether any adaptations are fidelity consistent (done to maintain core functions in our theory of change) or fidelity inconsistent (drifting away in a manner unlikely to maintain core functions), and whether proximal functions of the implementation (e.g., increased self-efficacy related to using guidelines).

#### Measures of fidelity to implementation elements

To index fidelity to implementation elements, school nurses and school personnel will answer questions about the completion and quality of each element:

*Adherence and adaptations* will be assessed using questions at T3 about training and support received during the study. *Satisfaction* will be assessed by asking how satisfied s/he was with the elements in SchoolHealth, and whether s/he would recommend them to a colleague. To assess *functions*, we will analyze the change in self-efficacy and collaboration adherence from pre to post. The school nurses will also be asked whether and how the elements helped them carry out the 8^th^-grade consultation and cooperate with schools. School personnel in element 3 will be asked whether and how the material helped them cooperate with the school health services and how many collaboration meetings they completed.

In addition, project coordinators register information about implementation in all experimental conditions. For training and consultations, the following will be registered: attendance, time spent, content completed, significant events, adaptations to plans, adherence. For technical assistance requested during the study that is of relevance to experimental conditions, the following will be registered: participant, time spent, content/issue, significant events, turnover/sick leaves, and other adaptations.

### Sustainment and scaling

Planning and preparing for sustainment and scale-up have been part of the co-creation process from the start of the exploration phase of the study. The projects' collaboration with key stakeholders, institutions educating health nurses, and authorities lays the foundation for using national infrastructure and regional competence centers (RBUP and RKBU) in scaling up.

The Norwegian Healthnet serves as a hub for developing a plan for sustainment and scale of functions in the DFA. This partnership provides a fruitful platform for designing, establishing, and testing secure data collection directly from users by means of Helsenorge.no, the digital platform for user interaction between citizens and patients with health services and registries.

Should the ongoing consultation (Dialog support) be a significant contributor to important implementation mechanisms and effects, we will plan for further improvements, sustainment, and scale by establishing an implementation group at the national competence centers involved in the study. Also, a protocol describing the structure, methods, and content of the ongoing consultations will be developed and made nationally available for other institutions to adopt. The e-learning module will be made accessible for educational purposes to the master's programs in public health nursing and will serve as a resource for the clinical practice of public health nursing.

If the results indicate that Collaboration material provides value, the material will be further improved based on participant feedback. RBUP and RKBU will offer schools and school health services an introduction and access to the revised material, which will be included as part of RBUP and RKBU Central Norway's ordinary teaching- and service provision.

In summary, each element in SchoolHealth can be sustained and scaled independently of the other, or in more ecological combinations. The results of the study will inform decisions regarding plans and recommendations for sustainment and scale.

### Dissemination of results

Results will be disseminated through scientific publications, the study's and collaborating institutions' webpages, seminars with school health services and schools, popular science publications, and press releases. Research fellows, who are part of the project team, will publish and publicly defend dissertations related to the study. Master students will also publish results from the study. Planned scientific publications include reporting results on primary outcomes, secondary outcomes, psychometrics, and implementation mechanisms. The project team determines authorship of scientific publications in line with the Vancouver Protocol.

## Discussion

This hybrid type 2 study can optimize large-scale strategies for implementing evidence-based guideline recommendations in school health services to improve students’ health literacy, positive health behaviors, identify students needing follow-up, and improve interprofessional collaboration. The study "deconstructs" a blended implementation strategy that has been co-created with a wide array of relevant stakeholders and partners into its smaller meaningful parts (i.e., implementation elements), which represents three human-centered discrete implementation strategies (audit and feedback + , ongoing consultations, and active dissemination). The multifactorial design allows testing the effects of the elements in isolation and all possible combinations, as well as testing hypothesized implementation mechanisms informed by theory. By combining methods from multiple paradigms (i.e., factorial design, pluralistic mixed-methods, phenomenology), we can investigate cause and effects, mechanisms, and value from the perspectives of complementary causal theories and the lived experience of participants. This will allow us also to explore narratives about how, when, and for whom value do or do not occur or emerge from the implementation strategies and use of guideline recommendations. The study also addresses the degree of guideline fidelity needed for intended effects to occur. Investigations as outlined above have been extensively called for to advance implementation science [7, 9, 61, 62].

The study evaluates an innovative digitalization effort co-developed to meet expressed needs of users and services. It will also extend knowledge on adolescents' service use and user-pathways important for developing youth-friendly human-centered models of primary care.

### Supplementary Information

**Additional file 1.** 

**Additional file 2.**

## Data Availability

Not applicable.

## Abbreviations

COM-B: Capabilities, Opportunities, and Motivation model of Behavior change
CONSORT: Consolidated Standards of Reporting Trials
DFA: Digital Feedback and Administration Tool
EPIS: Exploration, Preparation, Implementation, and Sustainment Framework
GSE-5: General Self-Efficacy, 5-item
KPR: The Norwegian Registry for Primary Health Care
NPR: Norwegian Patient Registry
NTNU: Norwegian University of Science and Technology
RBUP: The Centre for Child and Adolescent Mental Health, Eastern and Southern
RKBU: Regional Centre for Child and Youth Mental Health and Child Welfare
SDQ: The Strength and Difficulties Questionnaire
VID: VID Specialized University. The letters in VID are a Norwegian acronym for Specialized (Vitenskapelig), International (Internasjonal) and diaconal (diakonial)
Young-HUNT Study: The adolescent part of the Nord-Trøndelag Health Study
