# Supplementary material for: Guideline evaluation and implementation mechanisms in school health services (GuideMe): protocol for a hybrid randomized factorial trial
Source: BMC Health Serv Res. 2023 Nov 15;23:1259. doi: 10.1186/s12913-023-10179-2 (PMC10652429; doi:10.1186/s12913-023-10179-2)
Supplement: Supplementary file 1 — Additional file 1. [file 12913_2023_10179_MOESM1_ESM.docx]

**Supplementary file 1:**

**Outcome and measurement instrument information**

***The 8^th^ graders***

**The KIDSCREEN-27** (38) is a 27-item scale and will be used to assess generic health-related quality of life among the 8^th^ graders at T1 and T3. The scale has five dimensions: Physical Well-Being, Psychological Well-Being, Autonomy & Parents, Peers & Social Support and School Environment. Internal consistency values (Cronbach's Alpha) range between .79 (Physical Well-being) and .84 (Psychological Well-being) for the different dimensions for the self-report versions (56).

**The Strengths and difficulties questionnaire (SDQ)** (36) is a children self-report behavioural screening questionnaire, and will be used to measure prosocial, internalising and externalising behaviour among the 8^th^ graders at T1 and T3. The instrument includes 25 items that constitute 5 subscales: emotional symptoms (5 items), conduct problems (5 items), hyperactivity/inattention (5 items), peer relationship problems (5 items) and prosocial behaviour (5 items). In addition, the instrument includes a supplement with 6 items on the impact of the difficulties (36).

**Health Literacy for School-Aged Children (HLSAC)** (41) is a 10-item scale and will be used to assess health literacy among the 8^th^ grade students at T1, T2 and T3. The scale includes five core components – theoretical knowledge, practical knowledge, critical thinking, self-awareness, and citizenship – each component being tapped by two items. The instrument is constructed as a one-factor model. Psychometric measures have shown high Cronbach's alpha and good structural validity (confirmatory factor analysis). The instrument is not validated in Norwegian. The Norwegian translation was translated and re-translated from English by professional translators.

**Children's Somatic Symptoms Inventory (CSSI-8)** (37) will be used to measure nonspecific somatic symptoms among the 8^th^ graders at T1 and T2. Total scores are computed by summing the ratings for each item (0-4), in which higher scores indicate greater somatic distress. The instrument has shown good psychometric properties and is highly correlated with the 24-items version.

**General Self-Efficacy Scale, short version (GSE-5)** (39, 40) is a 5-item scale and will assess the self-efficacy of 8^th^ grade students at T1, T2 and T3. The scale is uni-dimensional and has shown good psychometric properties.

**User satisfaction** will be assessed by 8 items, covering user satisfaction and alliance with the school health nurse at T2. The items are partly self-developed, retrieved from different studies/surveys and slightly modified to fit the setting of the school health service. Four items are inspired by Haugum, Danielsen & Iversen (42), one item by Sjetne et. al (43) and one item is self-developed.

**School environment and attendance** are measured through a mix of self-developed questions and questions used in similar studies. Attendance is assessed using one question which maps the number of hours/days the student was absent in the last two weeks. Questions about bullying and conflicts, physical conditions at school, and student relations are adapted from Børnungeliv.dk, which is the Danish equivalent to the Digital dialog tool in SchoolHealth. Questions about exclusion and digital bullying is self-developed. Questions about the teacher-student relationship are inspired by a previous study about relationship-based class management (57).

***The school health nurses***

**Work-related Self-Efficacy (GSE-5)** (45) is a 5-item scale and will assess work-related self-efficacy in school health nurses at T1 and T3. This instrument is a revised version of General self-efficacy scale (GSE-5) (39, 40), adjusted to measure work-related self-efficacy.

**The Implementation Climate Scale (ICS)** (58) will be used to assess the strategic climate for implementation of guidelines in the participating school health services at T1 and T3. Separate versions for school health nurses and leaders will be completed. Psychometric properties have been found acceptable in different Norwegian health and welfare services (59, 60). Two subscales have been excluded due to incompatibility with school health services: the rewards subscale and the recognition subscale.

**The Implementation Leadership Scale (ILS)** (47), will be used to assess to what extent the leaders in the services support, and promote implementation of evidence-based interventions at T1 and T3. The psychometric properties have shown excellent internal consistency, and converging and diverging validity for the original version. Good psychometric properties have also been found in Norwegian mental health services (61).

**Implementability of guidelines** will be measured using the following three scales at T1 and T3: Feasibility of Intervention Measure (FIM), Acceptability of Intervention Measure (AIM), and Intervention Appropriateness Measure (IAM) (48). The measures have been translated to Norwegian and used in the child welfare services (25).

***School health nurses and their service leaders***

**Work-related Self-Efficacy (GSE-5)** (45) is a 5-item scale and will assess work-related self-efficacy at T1 and T2. This instrument is a revised version of General self-efficacy scale (GSE-5) (62), adjusted to measure work-related self-efficacy.

**The Implementation Climate Scale (ICS)** (58) will be used to assess the strategic climate for implementation of guidelines in the participating school health services at T1 and T3. Separate versions for school health nurses and leaders will be completed. Psychometric properties have been found acceptable in different Norwegian health and welfare services (59, 60). Two subscales have been excluded due to incompatibility with school health services: the rewards subscale and the recognition subscale.

**The Implementation Leadership Scale (ILS)** (47) will be used to assess to what extent the leaders in the services support, and promote implementation of evidence-based interventions at T1 and T3. The psychometric properties have shown excellent internal consistency and converging and diverging validity for the original version. Good psychometric properties have also been found in Norwegian mental health services (61).

**Implementability of guidelines** will be measured using the following at T1 and T3 three scales: Feasibility of Intervention Measure (FIM), Acceptability of Intervention Measure (AIM), and Intervention Appropriateness Measure (IAM) (48). The measures have been translated to Norwegian and used in the child welfare services (25) The measures are somewhat adjusted to fit the school health service.

***Leaders, teachers and school health nurses***

**Interprofessional collaboration** between the school and the school health services will be measured by a 8-item scale (44) at T1 and T3. The instrument is constructed as a one-factor model, with Cronbach's alpha .75. Small adjustments in the wording of some questions were done to make it more appropriate for the schools and school health services.

**Collaboration-adherence** is measured at T1 and T3 with a self-developed questionnaire (8 items) about how the collaboration is organized, whether school health nurses participated in any of the schools' planning hours or meetings, topics that the school and school health nurses collaborate on and how they collaborate.

**Collaboration between schools and the school health service.** Quality in collaboration is measured at T1 and T3 by 7 items measuring perceptions of the core functions of collaboration like: easy to contact each other, knowledge about each other's competence and tasks, mutual respect, structure etc.
