# Supplementary material for: Guideline evaluation and implementation mechanisms in school health services (GuideMe): protocol for a hybrid randomized factorial trial
Source: BMC Health Serv Res. 2023 Nov 15;23:1259. doi: 10.1186/s12913-023-10179-2 (PMC10652429; doi:10.1186/s12913-023-10179-2)
Supplement: Supplementary file 2 — Additional file 2. [file 12913_2023_10179_MOESM2_ESM.docx]

# Supplementary file 2:

## R-script – Power calculations

#Cluster represents school, and cluster size is average number og students.

#Factor is the three elements in SchoolHealth

#Uses R package "MOST"

install.packages("MOST")
library(MOST)

FactorialPowerPlan(assignment="between",

                   nfactors=3,

                   model_order=2,

                   d_main = .30,

                   cluster_size=30,

                   cluster_size_sd=15,

                   icc=.05,

                   power =.8)
